# Approaching Shared Pathophysiology in Immune-Mediated Diseases through Functional Genomics

**DOI:** 10.3390/genes11121482

**Published:** 2020-12-09

**Authors:** David González-Serna, Gonzalo Villanueva-Martin, Marialbert Acosta-Herrera, Ana Márquez, Javier Martín

**Affiliations:** 1 Institute of Parasitology and Biomedicine López-Neyra, Consejo Superior de Investigaciones Científicas (IPBLN-CSIC), 18016 Granada, Spain; sna.david@ipb.csic.es (D.G.-S.); gvillanuevamartin@ipb.csic.es (G.V.-M.); m.acostaherrera@ipb.csic.es (M.A.-H.); anamaort@ipb.csic.es (A.M.); 2 Systemic Autoimmune Disease Unit, Hospital Clínico San Cecilio, Instituto de Investigación Biosanitaria ibs.GRANADA, 18016 Granada, Spain

**Keywords:** functional genomics, immune-mediated diseases, trancriptomics, GWAS, Hi-C, shared genetics

## Abstract

Immune-mediated diseases (IMDs) are complex pathologies that are strongly influenced by environmental and genetic factors. Associations between genetic loci and susceptibility to these diseases have been widely studied, and hundreds of risk variants have emerged during the last two decades, with researchers observing a shared genetic pattern among them. Nevertheless, the pathological mechanism behind these associations remains a challenge that has just started to be understood thanks to functional genomic approaches. Transcriptomics, regulatory elements, chromatin interactome, as well as the experimental characterization of genomic findings, constitute key elements in the emerging understandings of how genetics affects the etiopathogenesis of IMDs. In this review, we will focus on the latest advances in the field of functional genomics, centering our attention on systemic rheumatic IMDs.

## 1. Introduction

Immune mediated diseases (IMDs) are a diverse group of pathologies with different etiologies, characterized by a dysregulation of the immune system. These diseases show different effects on the organism, including either systemic or local symptoms, which may overlap between the diseases [1]. This complexity makes their diagnosis a clinical challenge, as different IMDs are found to have shared comorbidities and may co-occur in the same patient. A common example is cardiovascular disorders, which are present in several of these diseases [2,3,4,5], as well as the presence of autoantibodies, which have great clinical and diagnostic significance [6,7].

Thus, the high rates of familial clustering and comorbidities observed across IMDs indicate that they share molecular mechanisms of disease pathogenesis [8]. In the last decades, large-scale genetic studies, such as genome-wide association studies (GWAS) and Immunochip studies [9,10] have been essential to our understanding of IMD genetics, allowing the identification of a considerable number of loci associated with each individual disease [11,12,13,14], but also suggesting the existence of a common genetic background in autoimmunity [8].

**Figure 1:**
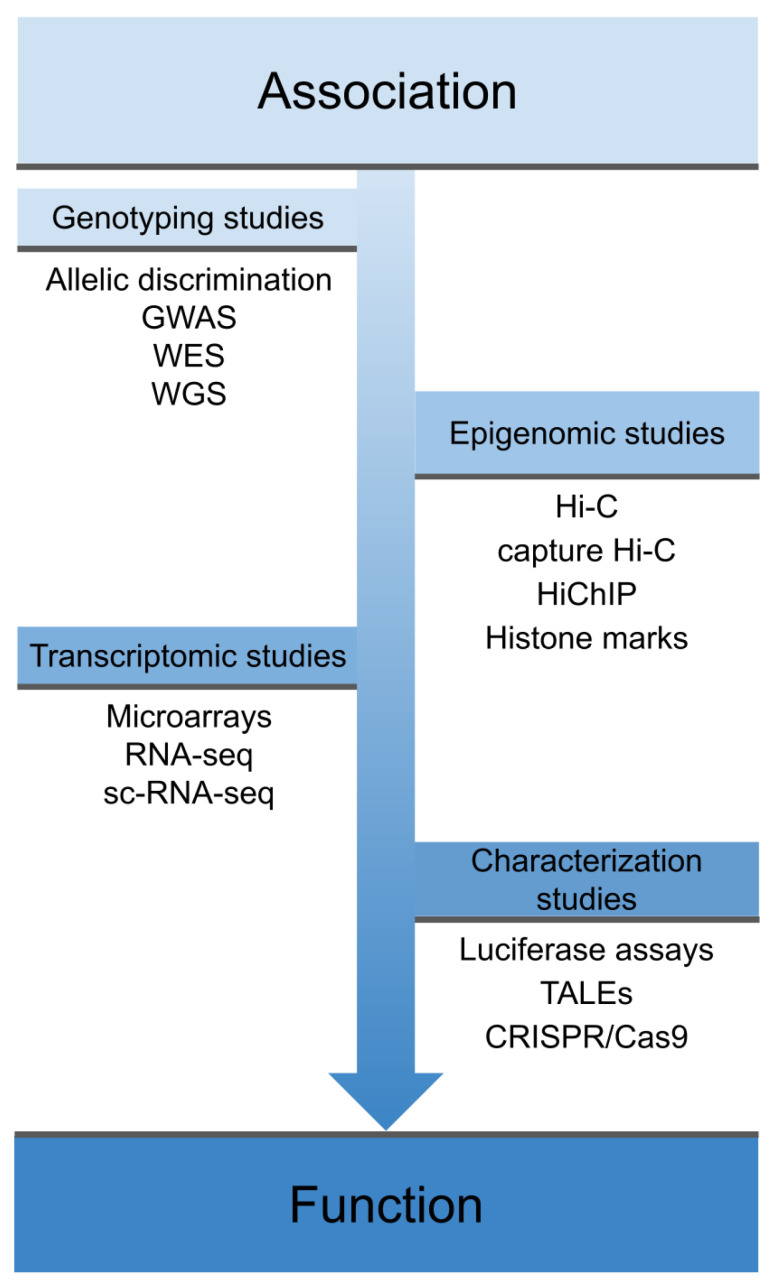
Overview of different techniques used in functional genomics. These techniques cover the spectrum from early association studies, through techniques that explore the physical interaction of these variants and their effects on the transcriptome, to phenotype characterization and gene function. GWAS: genome-wide association studies; WES: whole-exome sequencing; WGS: whole-genome sequencing; sc-RNA-seq: single-cell RNA sequencing, TALEs: Transcription activator-like effectors; CRISPR: clustered regularly interspaced short palindromic repeats; Cas9: CRISPR associated protein 9.

However, despite the success of GWAS, most of the polymorphisms associated with IMDs are located in non-coding regulatory regions of the genome and therefore their direct consequences on the disease are not clear [15,16]. In this regard, functional genomics is very useful in order to identify the mechanism of action of disease-associated variants and therefore the mechanisms underlying complex diseases, thus allowing progress in translating genetic findings to the clinic [17,18]. As shown in Figure 1, the process from the identification of a disease-associated variant to its characterization at the phenotypic level includes different functional assessments, which differ depending on the genomic location of the variant.

The purpose of this review is to highlight the latest advances in the field of functional genomics, with a particular focus on systemic rheumatic diseases.

## 2. Shared Genetics across IMDs

In recent years, many efforts have been made in order to identify risk loci shared among IMDs by combining GWAS or Immunochip data across several diseases. This strategy allows a direct comparison of the genetic component of these diseases, as well as an increase in statistical power to detect associations with low-effect variants. To date, a considerable amount of pairwise cross-disease meta-analyses of GWAS data of systemic rheumatic diseases has been published [19,20,21], which has led to the identification of many risk loci shared between pairs of these diseases. Furthermore, five studies combining the GWAS or Immunochip data of multiple IMDs simultaneously have been published, thus identifying a total of 75 new shared risk loci with some degree of pleiotropy in autoimmunity, which could partially explain the comorbidity observed among IMDs [22,23,24,25,26]. Good examples of known shared risk loci in IMDs are *PTPN22*, *IL23R* and *TNFAIP3*, which have allowed the repositioning of anti-TNF and anti-IL-12/IL-23 therapies to be used in rheumatoid arthritis (RA) and systemic lupus erythematosus (SLE), among others [1].

On the other hand, a recent study identified shared germline variants that predispose patients to RA, SLE and primary Sjögren’s syndrome (SjS) through whole-exome sequencing of 31 families, highlighting related T-cell-activating genes [27]. This familial aggregation suggests that a specific molecular pattern, leading to common pathogenesis in certain IMDs, could exist. Furthermore, Li et al. [22] quantified pairwise genetic sharing across 17 IMDs from the Immunobase resource, revealing a closer association among major systemic IMDs (including RA, SLE and systemic sclerosis (SSc)) than with other autoimmune disorders, such as psoriasis and inflammatory bowel disease. These studies support the idea that genetic pathways are shared among apparently clinically different IMDs and therefore a molecular reclassification of these diseases could lead to the discovery of new biomarkers for patient stratification and prognosis [28]. In line with this, a recent study stratified seven systemic IMDs into groups of molecular patterns, taking into account high dimensional molecular data including genome, transcriptome, and methylome data from whole blood samples, performing an unsupervised clustering analysis. Authors observed that systemic IMDs clustered in three different groups, representing “inflammatory”, “lymphoid” and “interferon” groups, with specific molecular patterns independently from their clinical classification [29].

The emergence of GWAS and associated genotyping platforms led to an increase in the number of variants associated with complex diseases, allowing for the development of more accurate genomic risk score (GRS) calculations, a direct application of genomic data to the clinical setting. GRS measures the additive effect of single nucleotide polymorphisms (SNPs), calculating the relative risk of individuals suffering from a given disease [30,31,32]. GRSs have been applied to different IMDs such as SLE, RA, SSc and psoriatic arthritis (PsA) [33,34,35,36]. Furthermore, genomic data can be useful for making a differential diagnosis, which is especially interesting in the case of inflammatory arthritis, since these conditions present with similar symptoms in the early stages [37]. In addition, the different relevant symptoms of the disease can be used to increase the predictive power of GRS, such as the appearance of lupus nephritis in SLE [33], or the appearance of autoantibodies in SSc [35].

## 3. Transcriptomic Studies

The analysis of the transcriptomic profile has been a major advance in genomics, especially thanks to the development of RNA sequencing (RNA-seq) technology [38], which has led to relevant findings in IMDs [39]. In this regard, transcriptomics has emerged as a useful approach to elucidate the target genes and pathways affected by genetic variants [40]. With GWAS, a global picture of the genetic variation across the genome influencing disease has been obtained. However, as previously mentioned, one of the main challenges in assigning biological meaning to GWAS findings is the fact that most disease-associated variants are located in non-coding regions of the genome. In recent years, it has been shown that these GWAS variants act as expression quantitative trait loci (eQTL), thus influencing disease by affecting gene expression levels through different mechanisms.

However, in the last few years, it has become clear that different cells show different gene expression profiles within a cell type, making it essential to evaluate the transcriptomic profile at single-cell resolution. The technique of single-cell RNA sequencing (scRNA-seq) [41,42,43] has made it possible to analyze the transcriptional profiles of key cells in the development of multiple and well-known diseases [44,45]. In this way, researchers have gone from studying large cell populations based on surface molecules to being able to differentiate cell subpopulations thanks to their gene expression profiles within a classical classification [46]. However, correctly identifying the true cell types present in a sample remains a challenge. In this regard, several bioinformatics tools have been recently developed in order to address this issue [47]. For example, some methods, such as that of Seurat [48] and Scanpy [49], allow for the clustering of subpopulations using the method of analyzing shared nearest neighbors, which mixes principal component analysis and graphical analysis.

Currently, there are no published studies of scRNA-seq among cross-diseases in IMDs, but given the relevance and impact of this technique; it is important to highlight existing studies in separate IMDs. In addition, it is expected that this novel approach will allow for the identification of common expression patterns and molecular pathways in autoimmunity. Several studies of scRNA-seq have been performed on immune cells of healthy controls compared against diseased cells. A signaling gradient, both spatial and transcriptional, was found for *NOTCH3* in perivascular fibroblasts from both RA patients and healthy controls, but they were upregulated in RA fibroblasts. Murine models showed a decrease in inflammation when deleting or blocking *Notch3* [50]. Furthermore, another study found that a subpopulation of fibroblasts—myofibroblasts, characterized by the presence of high expression levels of the *ACTA2* gene—were detected in fibroblasts from lung samples of healthy individuals and interstitial lung disease patients with SSc. This cellular subtype is considered to be a key player in the development of the disease, since myofibroblasts have a high capacity for synthesis and proliferation, and also produce very high levels of collagen type I. Therefore, this study suggests that *ACTA2* transcript levels could be used as a potential biomarker for SSc [51].

Several scRNA-seq studies have been performed in T-cells, a major cell type in the immune system with an important role in the pathogenesis of IMDs [52,53]. In order to search for biomarkers in RA, a recent study used scRNA-seq data to construct models of disease-associated cell types, their expression profiles and putative interactions using a multicellular model of disease. The authors observed that the analysis of the T-cell transcriptome can serve as a biomarker of different diseases, but also that the profile of T-cells can lead to the discovery of protein biomarkers in these cells [54].

Furthermore, an important application of this technique is the analysis of the B-cell receptor (BCR) and the T-cell receptor (TCR). These receptors, which are the result of the recombination of immunoglobulin genes during the development of these cells in the bone marrow, play a relevant role in autoimmunity [55,56,57]. The rise of high-throughput genotyping techniques and bioinformatic tools has allowed for the analysis of these highly variable and complex regions, such as BCR and TCR [58,59]. In this sense, islet antigen-reactive T-cell clonotypes were identified by their TCR profile in the peripheral blood of type 1 diabetes (T1D) patients [60]. A similar strategy was carried out in RA, in which the transcriptomic profile of the cell and the TCR of CD4+ T-cell clones was used, combining it with data on clonal expansion, tissue infiltration and membrane markers, enabling the detailed characterization of these cells [61].

Given the large amount of data being generated using different -omics approaches between different fields, their bioinformatics integration is crucial in order to achieve biological insights from genetics associations. An example is RolyPoly, which prioritizes disease-associated variants detected by GWAS using Bulk RNA-seq and scRNA-seq expression data [62].

## 4. Epigenomics Studies

Our knowledge of the chromatin landscape and the epigenetic regulatory elements, as well as their importance in disease pathogenesis, has undergone exponential growth in recent years. In this regard, different global initiatives and projects have emerged with the aim of sharing and putting together the knowledge in this area. Some of these projects are the Encyclopedia of DNA Elements (ENCODE) project [63], the Roadmap Epigenomics Consortium [64] and the Blueprint of Haematopoietic Epigenomes project [65], which aim to identify all the functional elements of the human genome across dozens of tissues.

Several epigenomic studies have been conducted in IMDs such as SSc or SjS in recent years, including histone modification, DNA methylation and non-coding RNA studies [66,67]. In this sense, a complex multidimensional study in RA fibroblast-like synoviocytes (FLS), an aggressive phenotype of fibroblasts in RA, was performed with the objective of establishing a high-resolution epigenomic landscape in RA. This study included histone modifications, open chromatin, RNA expression and DNA methylation analysis in RA FLS [68]. The effect of variants on histone regions is relevant, as seen in SLE. An enrichment of enhancer histone quantitative trait locus (hQTL) variants seems to have an important effect on gene expression in haplotypes with an eQTL in an SLE model, affecting it through the eQTL itself in most cases. Furthermore, its effect has been seen in HLA class II, when it is located in these regions, supporting data showing high allelic imbalance, even in the epigenome, in regions of post-translational histone modifications (PTM), using 3D chromatin techniques [69].

Another example of a multidimensional epigenomic study in IMDs is that performed in Takayasu arteritis, identifying the functional consequences of the susceptibility locus rs2069837 in a non-coding region of the *IL6*. Change in this region causes an alteration in a chromatin loop, leading to the recruitment of a repressor complex, which ends with the suppression of the anti-inflammatory gene *GPNMB* [70].

During the last years, our understanding of higher-order chromatin architecture has deeply changed. eQTL analyses have correlated genomic loci with variations in expression levels of nearby genes [71], thus providing a hypothetical mechanical link between a large number of loci associated with IMDs and the regulation of gene expression [72,73]. Nevertheless, little is known about how these genes and regulatory elements physically interact with each other across kilobase and megabase distances. In this sense, recent advances in 3D conformation of the genome can help to link local changes in chromatin with an effect on the expression of certain genes.

Nowadays, long-range chromatin interactions across an entire genome can be detected thanks to sequencing-based chromatin conformation capture techniques, highlighting Hi-C [74], which allows an ensemble average of interactions across the genome to be observed as a heatmap of interaction frequency. This spatial organization seems to be a general property of the genome, as it is consistent throughout and stable across different cell types, as well as being highly conserved between species. However, focusing at individual loci and at a higher resolution, more variability emerges. In this regard, a recent study performing Hi-C on lymphoblastoid cell lines from 20 individuals demonstrated that common SNPs can influence 3D chromatin conformation, and that this variation is often accompanied by variation in gene expression, transcription factor binding and histone modification [75].

In order to obtain high-resolution interaction maps, a new technique called capture Hi-C (CHi-C) enables an enrichment of Hi-C interactions through the capture of specific regions [76,77]. In this regard, Martin et al. characterized for the first time the interactions of confirmed susceptibility loci for four IMDs, namely RA, T1D, PsA and juvenile idiopathic arthritis (JIA) in different cell lines [76]. Interestingly, a considerable amount of disease-associated SNPs interacted with distant promoters instead of with the nearest gene, thus implicating different target genes. Subsequent studies using CHi-C data from T- and B-cell lines identified a novel causal gene, *IL20RA*, in the pan-autoimmune genetic susceptibility region 6q23 [77]. Associations within this region have been generally assigned to *TNFAIP3*, as it is the closest gene. Thus, these results provide new insights into IMD genetics, as well as a new perspective in the way we perceive the causal gene in disease, with the obvious implications that these findings have on pathogenic mechanisms and the discovery of potential therapeutic targets. In line with the above, a recently-published study performed CHi-C on RA-, JIA- and PsA-associated loci in T-helper and B-cell lines in order to identify high confidence candidate causal genes [78]. These genes were significantly enriched in disease-relevant pathways, and were used to interrogate drug target information to identify existing treatments that could be potentially repositioned to treat these diseases. In addition, the versatility of the CHi-C technique allows the identification of regions interacting with promoters (promoter CHi-C) [79,80]. In this regard, Burren et al. identified common candidate genes for five IMDs in activated T-cells using promoter CHi-C, highlighting an interaction-mediated regulation of *IL2RA* expression [81]. Furthermore, recently published results observed a specific promoter interaction landscape in THP-1 macrophages stimulated with lipopolysaccharide, with an enrichment of immune risk variants within the interacting regions [82]. These results demonstrate that the study of chromatin interactomes from specific cell types can help elucidate common patterns in regulatory pathways of IMDs.

A more recent technique called HiChIP has enabled the generation of contact maps combining Hi-C libraries with chromatin immunoprecipitation (ChIP), a technique used to investigate the interaction between proteins (such as transcription factors or determined histones) and DNA [83]. In this regard, a recent HiChIP study that combined the detection of H3K27ac and cohesin subunit Smc1 HiChIP in T-cells of T1D mice model showed hyperconnected 3D clusters in T1D-associated regions, including important genes in autoimmunity such as *BCL11B* and *ETS1*, whereas these hyperconnected clusters were not present in T-cells from diabetes-resistant mice [84]. Another study performed in immune cell line models used H3K27ac HiChIP in order to link SLE risk variants, traditionally associated with the autoimmunity risk gene *TNIP1*, with enhancer regions, observing that the *TNIP1* haplotype extended to neighboring genes [85].

The combination of the different techniques described above has led to the identification of relevant genes in autoimmune diseases. As an example, recent studies combining publicly available Hi-C, CHi-C and HiChIP data identified novel candidate genes for SSc and PsA [86].

## 5. Experimental Characterization

Despite major advances in functional genomics techniques, characterization studies remain the most successful tool to identify the effect of a mutation. In this way we can determine the cellular phenotype and are able to sort out the effects of suppressing, rescuing, over-expressing or under-expressing a gene in a controlled environment. There are different approaches in characterization studies, the most classic being fluorescent reporter studies (e.g., luciferase) and genome editing, such as transcription activator-like effectors (TALE) and the clustered regularly interspersed palindromic repeats (CRISPR) technique associated with Cas9 (CRISPR-Cas9).

Reporter studies have come a long way, with the development of high-throughput techniques using massive parallel reporter assays (MPRAs). An example is the association of non-coding candidate SNPs in several IMDs with the disruption of a regulatory circuit driven by NF-κB, which constrains T-cell activation through the upregulation of *TNFAIP3* (an inhibitor of NF-κB), thus leading to an autoimmune response in activated T-cells [87]. However, these techniques are still not as powerful as genetic editing studies, as they study variants outside their cellular and organic context. Among all the characterization tools, CRISPR-Cas9 stands out. This technique is based on the endonuclease repair capability of Cas9, and it is capable of making changes to the genome through genetic guidance [88].

Recent studies have demonstrated that this technology can be used to investigate and validate pathological mechanisms of IMDs proposed by the different genomic approaches previously mentioned. In this line, a recently published study targeted the variant rs6927172, which is strongly associated with some IMDs and is located upstream of the *TNFAIP3* gene. Using CRISPR/Cas9, the authors induced the deletion of this variant in HEK293 cells, resulting in reduced *TNFAIP3* expression, and, interestingly, they also found a reduction in *OLIG3* and *IL20RA* expression [89]. In a similar study performed in primary T-helper cells, researchers also observed a reduced expression of *TNFAIP3* after inducing the deletion of rs6927172, as well as other edits [87]. In addition, it is worth mentioning a CRISPR/Cas9 screening study developed in primary human T-cells in which the authors identified genes regulating the TCR response after stimulation [90]. The combination of CRISPR/Cas9 with other characterization and functional genomics techniques constitutes the perfect toolset to uncover mechanistic insights of potential functional variants. In this regard, a recent study combining CRISPR/Cas9 and luciferase assays with Hi-C and CHi-C data observed that the allele rs13239597-A, which is strongly associated with SSc and SLE, acts as an allele-specific enhancer regulating *IRF5* expression [91].

On the other hand, the CRISPR/Cas9 technology can also be used to directly treat IMDs. In a very interesting study, authors were able to activate the transcription of the insulin gene in fibroblasts from T1D patients using this technology [92]. Thus, more attention will be paid to CRISPR/Cas9 technology in the near future as a revolutionary method to study and treat IMDs.

## 6. New Approaches for Drug Targeting

The identification of new targets is a critical step in the drug discovery process. In this sense, the recent massive accumulation of different genomic data, mainly through GWAS, and their annotation through different functional data, establish the perfect framework to elucidate the underlying pathogenic mechanisms of IMDs and thus prioritize potential new therapeutic targets [93].

In this regard, one of the main examples of the usefulness of genomic studies in the identification of potential new drug targets is a study published by Okada et al. [11]. Through the largest GWAS conducted in RA, the authors identified more than 100 loci associated with this disorder. Subsequently, using bioinformatic methods based on functional annotation, they identified a total of 98 biological candidate genes, some of which were targets of RA therapies, whereas others were targets of drugs potentially useful for the treatment of this disease. A more recent GWAS performed in SSc identified a total of 27 independent risk loci for this condition. The subsequent functional analysis, using HiChIP data, of the most probable causal variants allowed the identification of 43 robust target genes, highlighting *CD80* and *BLK* as potential drug targets [14]. Furthermore, meta-analysis of GWAS data, including different IMDs, also led to the identification of shared risk loci, as well as potential new drug targets through drug enrichment analysis [24,26]. It is worth mentioning that drugs with mechanisms based on genetic evidence have a higher probability to be approved through the drug development process [94].

On the other hand, the biological function of a genetic change can be easy to observe, thus making it easy to identify a potential drug target. For example, the knowledge of mutations and deletions occurring in the *JAK3* gene that cause severe combined immunodeficiency [95] was useful to develop drugs such as tofacitinib to treat inflammation in RA [96].

Additionally, genomic and epigenomic data, using functional genomic approaches, are being integrated in order to facilitate drug repurposing in IMDs [97]. In this regard, analysis of capture Hi-C data from B-cells and T-cells to identify causal genes for rheumatic diseases revealed many disease-associated genes to be targets of existing drugs [78]. A recent study demonstrated an approach that integrates functional genomics and immune-mediated annotations with the evidence of physical interaction in order to prioritize drug targets, an approach which has been validated and applied in RA [98]. In this sense, the emergence of TNF as a highly ranked target confirms the utility of this approach [98,99].

## 7. Conclusions

As noted throughout this review, the combination of different functional genomics approaches is proving useful for the identification of potentially causal variants and causal genes in autoimmunity. In this regard, the whole process, from the early discovery of genetic associations through association studies to the validation and functional repercussions of these signals, has become an essential and holistic strategy (Figure 1). Today, it is evident that these approaches that in the past were applied separately must be taken from a global point of view in order to fully understand the underlying pathological mechanisms of IMDs. The reduction in the cost of these techniques has allowed an increasing number of researchers to afford to use a combination of these experiments to refine associations with IMD susceptibility. Thus, the genetic background of IMDs and their manifestation through their comorbidities is likely to lead to the discovery of shared genetic mechanisms. Elucidating the key features of the genetic changes shared by these diseases will allow a better classification of patients, as well as the development of more effective therapeutic interventions.

## References

[1] Cho J.H., Feldman M. (2015). Heterogeneity of autoimmune diseases: Pathophysiologic insights from genetics and implications for new therapies. Nat. Med..

[2] López-Mejías R., Castañeda S., González-Juanatey C., Corrales A., Ferraz-Amaro I., Genre F., Remuzgo-Martínez S., Rodriguez-Rodriguez L., Blanco R., Llorca J. (2016). Cardiovascular risk assessment in patients with rheumatoid arthritis: The relevance of clinical, genetic and serological markers. Autoimmun. Rev..

[3] Lisnevskaia L., Murphy G., Isenberg D. (2014). Systemic lupus erythematosus. Lancet.

[4] Denton C.P., Khanna D. (2017). Systemic sclerosis. Lancet.

[5] Smolen J.S., Aletaha D., McInnes I.B. (2016). Rheumatoid arthritis. Lancet.

[6] Pisetsky D.S., Lipsky P.E. (2020). New insights into the role of antinuclear antibodies in systemic lupus erythematosus. Nat. Rev. Rheumatol..

[7] Bournia V.-K., Vlachoyiannopoulos P.G. (2012). Subgroups of Sjögren syndrome patients according to serological profiles. J. Autoimmun..

[8] Parkes M., Cortes A., van Heel D.A., Brown M.A. (2013). Genetic insights into common pathways and complex relationships among immune-mediated diseases. Nat. Rev. Genet..

[9] Polychronakos C. (2011). Fine points in mapping autoimmunity. Nat. Genet..

[10] Cortes A., Brown M.A. (2011). Promise and pitfalls of the Immunochip. Arthritis Res. Ther..

[11] Okada Y., Wu D., Trynka G., Raj T., Terao C., Ikari K., Kochi Y., Ohmura K., Suzuki A., Yoshida S. (2014). Genetics of rheumatoid arthritis contributes to biology and drug discovery. Nature.

[12] Bentham J., Morris D.L., Cunninghame Graham D.S., Pinder C.L., Tombleson P., Behrens T.W., Martín J., Fairfax B.P., Knight J.C., Chen L. (2015). Genetic association analyses implicate aberrant regulation of innate and adaptive immunity genes in the pathogenesis of systemic lupus erythematosus. Nat. Genet..

[13] Carmona F.D., Vaglio A., Mackie S.L., Hernández-Rodríguez J., Monach P.A., Castañeda S., Solans R., Morado I.C., Narváez J., Ramentol-Sintas M. (2017). A genome-wide association study identifies risk alleles in plasminogen and P4HA2 associated with giant cell arteritis. Am. J. Hum. Genet..

[14] López-Isac E., Acosta-Herrera M., Kerick M., Assassi S., Satpathy A.T., Granja J., Mumbach M.R., Beretta L., Simeón C.P., Carreira P. (2019). GWAS for systemic sclerosis identifies multiple risk loci and highlights fibrotic and vasculopathy pathways. Nat. Commun..

[15] Visscher P.M., Wray N.R., Zhang Q., Sklar P., McCarthy M.I., Brown M.A., Yang J. (2017). 10 years of GWAS discovery: Biology, function, and translation. Am. J. Hum. Genet..

[16] Gallagher M.D., Chen-Plotkin A.S. (2018). The post-GWAS era: From association to function. Am. J. Hum. Genet..

[17] Ding J., Frantzeskos A., Orozco G. (2020). Functional genomics in autoimmune diseases. Hum. Mol. Genet..

[18] Zeggini E., Gloyn A.L., Barton A.C., Wain L.V. (2019). Translational genomics and precision medicine: Moving from the lab to the clinic. Science.

[19] Martin J.-E., Assassi S., Diaz-Gallo L.-M., Broen J.C., Simeon C.P., Castellvi I., Vicente-Rabaneda E., Fonollosa V., Ortego-Centeno N., González-Gay M.A. (2013). A systemic sclerosis and systemic lupus erythematosus pan-meta-GWAS reveals new shared susceptibility loci. Hum. Mol. Genet..

[20] López-Isac E., Martín J.-E., Assassi S., Simeón C.P., Carreira P., Ortego-Centeno N., Freire M., Beltrán E., Narváez J., Alegre-Sancho J.J. (2016). Brief report: IRF4 newly identified as a common susceptibility locus for systemic sclerosis and rheumatoid arthritis in a cross-disease meta-analysis of genome-wide association studies. Arthritis Rheumatol..

[21] Márquez A., Vidal-Bralo L., Rodríguez-Rodríguez L., González-Gay M.A., Balsa A., González-Álvaro I., Carreira P., Ortego-Centeno N., Ayala-Gutiérrez M.M., García-Hernández F.J. (2017). A combined large-scale meta-analysis identifies COG6 as a novel shared risk locus for rheumatoid arthritis and systemic lupus erythematosus. Ann. Rheum. Dis..

[22] Li Y.R., Li J., Zhao S.D., Bradfield J.P., Mentch F.D., Maggadottir S.M., Hou C., Abrams D.J., Chang D., Gao F. (2015). Meta-analysis of shared genetic architecture across ten pediatric autoimmune diseases. Nat. Med..

[23] Ellinghaus D., Jostins L., Spain S.L., Cortes A., Bethune J., Han B., Park Y.R., Raychaudhuri S., Pouget J.G., Hübenthal M. (2016). Analysis of five chronic inflammatory diseases identifies 27 new associations and highlights disease-specific patterns at shared loci. Nat. Genet..

[24] Márquez A., Kerick M., Zhernakova A., Gutierrez-Achury J., Chen W.-M., Onengut-Gumuscu S., González-Álvaro I., Rodriguez-Rodriguez L., Rios-Fernández R., González-Gay M.A. (2018). Meta-analysis of immunochip data of four autoimmune diseases reveals novel single-disease and cross-phenotype associations. Genome Med..

[25] Ortiz-Fernández L., Carmona F.D., López-Mejías R., González-Escribano M.F., Lyons P.A., Morgan A.W., Sawalha A.H., Merkel P.A., Smith K.G.C., González-Gay M.A. (2018). Cross-phenotype analysis of immunochip data identifies KDM4C as a relevant locus for the development of systemic vasculitis. Ann. Rheum. Dis..

[26] Acosta-Herrera M., Kerick M., González-Serna D., Wijmenga C., Franke A., Gregersen P.K., Padyukov L., Worthington J., Vyse T.J., Alarcón-Riquelme M.E. (2019). Genome-wide meta-analysis reveals shared new loci in systemic seropositive rheumatic diseases. Ann. Rheum. Dis..

[27] Wang Y., Chen S., Chen J., Xie X., Gao S., Zhang C., Zhou S., Wang J., Mai R., Lin Q. (2020). Germline genetic patterns underlying familial rheumatoid arthritis, systemic lupus erythematosus and primary Sjögren’s syndrome highlight T cell-initiated autoimmunity. Ann. Rheum. Dis..

[28] Barturen G., Beretta L., Cervera R., Van Vollenhoven R., Alarcón-Riquelme M.E. (2018). Moving towards a molecular taxonomy of autoimmune rheumatic diseases. Nat. Rev. Rheumatol..

[29] Barturen G., Babaei S., Català-Moll F., Martínez-Bueno M., Makowska Z., Martorell-Marugán J., Carmona-Sáez P., Toro-Domínguez D., Carnero-Montero E., Teruel M. (2020). Integrative Analysis Reveals a Molecular Stratification of Systemic Autoimmune Diseases. Arthritis Rheumatol..

[30] Khera A.V., Chaffin M., Aragam K.G., Haas M.E., Roselli C., Choi S.H., Natarajan P., Lander E.S., Lubitz S.A., Ellinor P.T. (2018). Genome-wide polygenic scores for common diseases identify individuals with risk equivalent to monogenic mutations. Nat. Genet..

[31] Torkamani A., Wineinger N.E., Topol E.J. (2018). The personal and clinical utility of polygenic risk scores. Nat. Rev. Genet..

[32] Choi S.W., Mak T.S.-H., O’Reilly P.F. (2020). Tutorial: A guide to performing polygenic risk score analyses. Nat. Protoc..

[33] Chen L., Wang Y.-F., Liu L., Bielowka A., Ahmed R., Zhang H., Tombleson P., Roberts A.L., Odhams C.A., Cunninghame Graham D.S. (2020). Genome-wide assessment of genetic risk for systemic lupus erythematosus and disease severity. Hum. Mol. Genet..

[34] Stahl E.A., Wegmann D., Trynka G., Gutierrez-Achury J., Do R., Voight B.F., Kraft P., Chen R., Kallberg H.J., Kurreeman F.A.S. (2012). Bayesian inference analyses of the polygenic architecture of rheumatoid arthritis. Nat. Genet..

[35] Bossini-Castillo L., Villanueva-Martin G., Kerick M., Acosta-Herrera M., López-Isac E., Simeón C.P., Ortego-Centeno N., Assassi S., International SSc Group, Australian Scleroderma Interest Group (ASIG) (2020). Genomic risk score impact on susceptibility to systemic sclerosis. Ann. Rheum. Dis..

[36] Patrick M.T., Stuart P.E., Raja K., Gudjonsson J.E., Tejasvi T., Yang J., Chandran V., Das S., Callis-Duffin K., Ellinghaus E. (2018). Genetic signature to provide robust risk assessment of psoriatic arthritis development in psoriasis patients. Nat. Commun..

[37] Knevel R., le Cessie S., Terao C.C., Slowikowski K., Cui J., Huizinga T.W.J., Costenbader K.H., Liao K.P., Karlson E.W., Raychaudhuri S. (2020). Using genetics to prioritize diagnoses for rheumatology outpatients with inflammatory arthritis. Sci. Transl. Med..

[38] Wang Z., Gerstein M., Snyder M. (2009). RNA-Seq: A revolutionary tool for transcriptomics. Nat. Rev. Genet..

[39] Banchereau R., Cepika A.-M., Banchereau J., Pascual V. (2017). Understanding human autoimmunity and autoinflammation through transcriptomics. Annu. Rev. Immunol..

[40] Ricaño-Ponce I., Zhernakova D.V., Deelen P., Luo O., Li X., Isaacs A., Karjalainen J., Di Tommaso J., Borek Z.A., Zorro M.M. (2016). Refined mapping of autoimmune disease associated genetic variants with gene expression suggests an important role for non-coding RNAs. J. Autoimmun..

[41] Kurimoto K., Yabuta Y., Ohinata Y., Ono Y., Uno K.D., Yamada R.G., Ueda H.R., Saitou M. (2006). An improved single-cell cDNA amplification method for efficient high-density oligonucleotide microarray analysis. Nucleic Acids Res..

[42] Kurimoto K., Yabuta Y., Ohinata Y., Saitou M. (2007). Global single-cell cDNA amplification to provide a template for representative high-density oligonucleotide microarray analysis. Nat. Protoc..

[43] Tang F., Barbacioru C., Wang Y., Nordman E., Lee C., Xu N., Wang X., Bodeau J., Tuch B.B., Siddiqui A. (2009). mRNA-Seq whole-transcriptome analysis of a single cell. Nat. Methods.

[44] Suvà M.L., Tirosh I. (2019). Single-Cell RNA sequencing in cancer: Lessons learned and emerging challenges. Mol. Cell.

[45] Paik D.T., Cho S., Tian L., Chang H.Y., Wu J.C. (2020). Single-cell RNA sequencing in cardiovascular development, disease and medicine. Nat. Rev. Cardiol..

[46] Altschuler S.J., Wu L.F. (2010). Cellular heterogeneity: Do differences make a difference?. Cell.

[47] Kiselev V.Y., Andrews T.S., Hemberg M. (2019). Challenges in unsupervised clustering of single-cell RNA-seq data. Nat. Rev. Genet..

[48] Stegle O., Teichmann S.A., Marioni J.C. (2015). Computational and analytical challenges in single-cell transcriptomics. Nat. Rev. Genet..

[49] Wolf F.A., Angerer P., Theis F.J. (2018). SCANPY: Large-scale single-cell gene expression data analysis. Genome Biol..

[50] Wei K., Korsunsky I., Marshall J.L., Gao A., Watts G.F.M., Major T., Croft A.P., Watts J., Blazar P.E., Lange J.K. (2020). Notch signalling drives synovial fibroblast identity and arthritis pathology. Nature.

[51] Valenzi E., Bulik M., Tabib T., Morse C., Sembrat J., Trejo Bittar H., Rojas M., Lafyatis R. (2019). Single-cell analysis reveals fibroblast heterogeneity and myofibroblasts in systemic sclerosis-associated interstitial lung disease. Ann. Rheum. Dis..

[52] Orvain C., Assassi S., Avouac J., Allanore Y. (2020). Systemic sclerosis pathogenesis: Contribution of recent advances in genetics. Curr. Opin. Rheumatol..

[53] Firestein G.S., McInnes I.B. (2017). Immunopathogenesis of rheumatoid arthritis. Immunity.

[54] Gawel D.R., Serra-Musach J., Lilja S., Aagesen J., Arenas A., Asking B., Bengnér M., Björkander J., Biggs S., Ernerudh J. (2019). A validated single-cell-based strategy to identify diagnostic and therapeutic targets in complex diseases. Genome Med..

[55] Wardemann H., Yurasov S., Schaefer A., Young J.W., Meffre E., Nussenzweig M.C. (2003). Predominant autoantibody production by early human B cell precursors. Science.

[56] Stavnezer J., Schrader C.E. (2014). IgH chain class switch recombination: Mechanism and regulation. J. Immunol..

[57] Bashford-Rogers R.J.M., Bergamaschi L., McKinney E.F., Pombal D.C., Mescia F., Lee J.C., Thomas D.C., Flint S.M., Kellam P., Jayne D.R.W. (2019). Analysis of the B cell receptor repertoire in six immune-mediated diseases. Nature.

[58] Singh M., Al-Eryani G., Carswell S., Ferguson J.M., Blackburn J., Barton K., Roden D., Luciani F., Phan T.G., Junankar S. (2019). High-throughput targeted long-read single cell sequencing reveals the clonal and transcriptional landscape of lymphocytes. Nat. Commun..

[59] Van Eerden S.L., Standley D.M., Teraguchi S., Saputri D.S., Llamas-Covarrubias M.A., Davila A., Diez D., Nazlica S.A., Rozewicki J., Ismanto H.S. (2020). Methods for sequence and structural analysis of B and T cell receptor repertoires. Comput. Struct. Biotechnol. J..

[60] Cerosaletti K., Barahmand-pour-Whitman F., Yang J., DeBerg H.A., Dufort M.J., Murray S.A., Israelsson E., Speake C., Gersuk V.H., Eddy J.A. (2017). Single-cell RNA sequencing reveals expanded clones of islet antigen-reactive CD4 T cells in peripheral blood of subjects with type 1 diabetes. J. Immunol..

[61] Ishigaki K., Shoda H., Kochi Y., Yasui T., Kadono Y., Tanaka S., Fujio K., Yamamoto K. (2015). Quantitative and qualitative characterization of expanded CD4+ T cell clones in rheumatoid arthritis patients. Sci. Rep..

[62] Calderon D., Bhaskar A., Knowles D.A., Golan D., Raj T., Fu A.Q., Pritchard J.K. (2017). Inferring relevant cell types for complex traits by using single-cell gene expression. Am. J. Hum. Genet..

[63] (2004). ENCODE Project Consortium The ENCODE (ENCyclopedia of DNA elements) project. Science.

[64] Kundaje A., Meuleman W., Ernst J., Bilenky M., Yen A., Heravi-Moussavi A., Kheradpour P., Zhang Z., Wang J., Roadmap Epigenomics Consortium (2015). Integrative analysis of 111 reference human epigenomes. Nature.

[65] Fernández J.M., de la Torre V., Richardson D., Royo R., Puiggròs M., Moncunill V., Fragkogianni S., Clarke L., Flicek P., BLUEPRINT Consortium (2016). The BLUEPRINT data analysis portal. Cell Syst..

[66] Angiolilli C., Marut W., van der Kroef M., Chouri E., Reedquist K.A., Radstake T.R.D.J. (2018). New insights into the genetics and epigenetics of systemic sclerosis. Nat. Rev. Rheumatol..

[67] Imgenberg-Kreuz J., Sandling J.K., Nordmark G. (2018). Epigenetic alterations in primary Sjögren’s syndrome—An overview. Clin. Immunol..

[68] Tsuchiya H., Ota M., Sumitomo S., Ishigaki K., Suzuki A., Sakata T., Tsuchida Y., Inui H., Hirose J., Kochi Y. (2020). Parsing multiomics landscape of activated synovial fibroblasts highlights drug targets linked to genetic risk of rheumatoid arthritis. Ann. Rheum. Dis..

[69] Pelikan R.C., Kelly J.A., Fu Y., Lareau C.A., Tessneer K.L., Wiley G.B., Wiley M.M., Glenn S.B., Harley J.B., Guthridge J.M. (2018). Enhancer histone-QTLs are enriched on autoimmune risk haplotypes and influence gene expression within chromatin networks. Nat. Commun..

[70] Kong X., Sawalha A.H. (2019). Takayasu arteritis risk locus in represses the anti-inflammatory gene through chromatin looping and recruiting MEF2-HDAC complex. Ann. Rheum. Dis..

[71] Lappalainen T., Sammeth M., Friedländer M.R., ’t Hoen P.A.C., Monlong J., Rivas M.A., Gonzàlez-Porta M., Kurbatova N., Griebel T., Ferreira P.G. (2013). Transcriptome and genome sequencing uncovers functional variation in humans. Nature.

[72] Westra H.-J., Peters M.J., Esko T., Yaghootkar H., Schurmann C., Kettunen J., Christiansen M.W., Fairfax B.P., Schramm K., Powell J.E. (2013). Systematic identification of trans eQTLs as putative drivers of known disease associations. Nat. Genet..

[73] Odhams C.A., Cunninghame Graham D.S., Vyse T.J. (2017). Profiling RNA-Seq at multiple resolutions markedly increases the number of causal eQTLs in autoimmune disease. PLoS Genet..

[74] Lieberman-Aiden E., van Berkum N.L., Williams L., Imakaev M., Ragoczy T., Telling A., Amit I., Lajoie B.R., Sabo P.J., Dorschner M.O. (2009). Comprehensive mapping of long-range interactions reveals folding principles of the human genome. Science.

[75] Gorkin D.U., Qiu Y., Hu M., Fletez-Brant K., Liu T., Schmitt A.D., Noor A., Chiou J., Gaulton K.J., Sebat J. (2019). Common DNA sequence variation influences 3-dimensional conformation of the human genome. Genome Biol..

[76] Martin P., McGovern A., Orozco G., Duffus K., Yarwood A., Schoenfelder S., Cooper N.J., Barton A., Wallace C., Fraser P. (2015). Capture Hi-C reveals novel candidate genes and complex long-range interactions with related autoimmune risk loci. Nat. Commun..

[77] McGovern A., Schoenfelder S., Martin P., Massey J., Duffus K., Plant D., Yarwood A., Pratt A.G., Anderson A.E., Isaacs J.D. (2016). Capture Hi-C identifies a novel causal gene, IL20RA, in the pan-autoimmune genetic susceptibility region 6q23. Genome Biol..

[78] Martin P., Ding J., Duffus K., Gaddi V.P., McGovern A., Ray-Jones H., Yarwood A., Worthington J., Barton A., Orozco G. (2019). Chromatin interactions reveal novel gene targets for drug repositioning in rheumatic diseases. Ann. Rheum. Dis..

[79] Schoenfelder S., Furlan-Magaril M., Mifsud B., Tavares-Cadete F., Sugar R., Javierre B.-M., Nagano T., Katsman Y., Sakthidevi M., Wingett S.W. (2015). The pluripotent regulatory circuitry connecting promoters to their long-range interacting elements. Genome Res..

[80] Javierre B.M., Burren O.S., Wilder S.P., Kreuzhuber R., Hill S.M., Sewitz S., Cairns J., Wingett S.W., Várnai C., Thiecke M.J. (2016). Lineage-specific genome architecture links enhancers and non-coding disease variants to target gene promoters. Cell.

[81] Burren O.S., Rubio García A., Javierre B.-M., Rainbow D.B., Cairns J., Cooper N.J., Lambourne J.J., Schofield E., Castro Dopico X., Ferreira R.C. (2017). Chromosome contacts in activated T cells identify autoimmune disease candidate genes. Genome Biol..

[82] Pradhananga S., Spalinskas R., Poujade F.-A., Eriksson P., Sahlén P. (2020). Promoter anchored interaction landscape of THP-1 macrophages captures early immune response processes. Cell. Immunol..

[83] Mumbach M.R., Satpathy A.T., Boyle E.A., Dai C., Gowen B.G., Cho S.W., Nguyen M.L., Rubin A.J., Granja J.M., Kazane K.R. (2017). Enhancer connectome in primary human cells identifies target genes of disease-associated DNA elements. Nat. Genet..

[84] Fasolino M., Goldman N., Wang W., Cattau B., Zhou Y., Petrovic J., Link V.M., Cote A., Chandra A., Silverman M. (2020). Genetic variation in type 1 diabetes reconfigures the 3D chromatin organization of T cells and alters gene expression. Immunity.

[85] Pasula S., Tessneer K.L., Fu Y., Gopalakrishnan J., Pelikan R.C., Kelly J.A., Wiley G.B., Wiley M.M., Gaffney P.M. (2020). Role of systemic lupus erythematosus risk variants with opposing functional effects as a driver of hypomorphic expression of TNIP1 and other genes within a three-dimensional chromatin network. Arthritis Rheumatol..

[86] Shi C., Ray-Jones H., Ding J., Duffus K., Fu Y., Gaddi V.P., Gough O., Hankinson J., Martin P., McGovern A. (2020). An active chromatin interactome in relevant cell lines elucidates biological mechanisms at genetic risk loci for dermatological traits. BioRxiv.

[87] Bourges C., Groff A.F., Burren O.S., Gerhardinger C., Mattioli K., Hutchinson A., Hu T., Anand T., Epping M.W., Wallace C. (2020). Resolving mechanisms of immune-mediated disease in primary CD4 T cells. EMBO Mol. Med..

[88] Ran F.A., Hsu P.D., Wright J., Agarwala V., Scott D.A., Zhang F. (2013). Genome engineering using the CRISPR-Cas9 system. Nat. Protoc..

[89] Wu J., Yang S., Yu D., Gao W., Liu X., Zhang K., Fu X., Bao W., Zhang K., Yu J. (2018). CRISPR/cas9 mediated knockout of an intergenic variant rs6927172 identified IL-20RA as a new risk gene for multiple autoimmune diseases. Genes Immun..

[90] Shifrut E., Carnevale J., Tobin V., Roth T.L., Woo J.M., Bui C.T., Li P.J., Diolaiti M.E., Ashworth A., Marson A. (2018). Genome-wide CRISPR screens in primary human T cells reveal key regulators of immune function. Cell.

[91] Thynn H.N., Chen X.-F., Hu W.-X., Duan Y.-Y., Zhu D.-L., Chen H., Wang N.-N., Chen H.-H., Rong Y., Lu B.-J. (2020). An allele-specific functional SNP associated with two systemic autoimmune diseases modulates IRF5 expression by long-range chromatin loop formation. J. Investig. Dermatol..

[92] Giménez C.A., Ielpi M., Mutto A., Grosembacher L., Argibay P., Pereyra-Bonnet F. (2016). CRISPR-on system for the activation of the endogenous human INS gene. Gene Ther..

[93] Finan C., Gaulton A., Kruger F.A., Lumbers R.T., Shah T., Engmann J., Galver L., Kelley R., Karlsson A., Santos R. (2017). The druggable genome and support for target identification and validation in drug development. Sci. Transl. Med..

[94] Nelson M.R., Tipney H., Painter J.L., Shen J., Nicoletti P., Shen Y., Floratos A., Sham P.C., Li M.J., Wang J. (2015). The support of human genetic evidence for approved drug indications. Nat. Genet..

[95] Russell S.M., Tayebi N., Nakajima H., Riedy M.C., Roberts J.L., Aman M.J., Migone T.-S., Noguchi M., Markert M.L., Buckley R.H. (1995). Mutation of Jak3 in a patient with SCID: Essential role of jak3 in lymphoid development. Science.

[96] Van Vollenhoven R.F., Fleischmann R., Cohen S., Lee E.B., García Meijide J.A., Wagner S., Forejtova S., Zwillich S.H., Gruben D., Koncz T. (2012). Tofacitinib or adalimumab versus placebo in rheumatoid arthritis. N. Engl. J. Med..

[97] Kingsmore K.M., Grammer A.C., Lipsky P.E. (2020). Drug repurposing to improve treatment of rheumatic autoimmune inflammatory diseases. Nat. Rev. Rheumatol..

[98] Fang H., De Wolf H., Knezevic B., Burnham K.L., Osgood J., Sanniti A., LledóLara A., Kasela S., De Cesco S., ULTRA-DD Consortium (2019). A genetics-led approach defines the drug target landscape of 30 immune-related traits. Nat. Genet..

[99] Fang H., Chen L., Knight J.C. (2020). From genome-wide association studies to rational drug target prioritisation in inflammatory arthritis. Lancet Rheumatol..

